# Fast contour propagation for MR‐guided prostate radiotherapy using convolutional neural networks

**DOI:** 10.1002/mp.13994

**Published:** 2020-01-23

**Authors:** K.A.J. Eppenhof, M. Maspero, M.H.F. Savenije, J.C.J. de Boer, J.R.N. van der Voort van Zyp, B.W. Raaymakers, A.J.E. Raaijmakers, M. Veta, C.A.T. van den Berg, J.P.W. Pluim

**Affiliations:** ^1^ Medical Image Analysis Group, Department of Biomedical Engineering Eindhoven University of Technology Eindhoven The Netherlands; ^2^ Computational Imaging Group for MR Diagnostics & Therapy, Center for Image Sciences University Medical Center Utrecht Utrecht The Netherlands; ^3^ Department of Radiotherapy University Medical Center Utrecht Utrecht The Netherlands; ^4^ Image Sciences Institute University Medical Center Utrecht Utrecht The Netherlands

**Keywords:** contour propagation, deep learning, image registration, MR-guided radiotherapy, prostate

## Abstract

**Purpose:**

To quickly and automatically propagate organ contours from pretreatment to fraction images in magnetic resonance (MR)‐guided prostate external‐beam radiotherapy.

**Methods:**

Five prostate cancer patients underwent 20 fractions of image‐guided external‐beam radiotherapy on a 1.5 T MR‐Linac system. For each patient, a pretreatment T2‐weighted three‐dimensional (3D) MR imaging (MRI) scan was used to delineate the clinical target volume (CTV) contours. The same scan was repeated during each fraction, with the CTV contour being manually adapted if necessary. A convolutional neural network (CNN) was trained for combined image registration and contour propagation. The network estimated the propagated contour and a deformation field between the two input images. The training set consisted of a synthetically generated ground truth of randomly deformed images and prostate segmentations. We performed a leave‐one‐out cross‐validation on the five patients and propagated the prostate segmentations from the pretreatment to the fraction scans. Three variants of the CNN, aimed at investigating supervision based on optimizing segmentation overlap, optimizing the registration, and a combination of the two were compared to results of the open‐source deformable registration software package Elastix.

**Results:**

The neural networks trained on segmentation overlap or the combined objective achieved significantly better Hausdorff distances between predicted and ground truth contours than Elastix, at the much faster registration speed of 0.5 s. The CNN variant trained to optimize both the prostate overlap and deformation field, and the variant trained to only maximize the prostate overlap, produced the best propagation results.

**Conclusions:**

A CNN trained on maximizing prostate overlap and minimizing registration errors provides a fast and accurate method for deformable contour propagation for prostate MR‐guided radiotherapy.

## 1 Introduction

External‐beam radiotherapy is one of the standard treatments for prostate cancer.^1^ Because of the superior soft‐tissue contrast, magnetic resonance imaging (MRI) is increasingly used in planning and guiding prostate radiotherapy.^2^, ^3^ Extreme hypofractionation with stereotactic body radiotherapy (SBRT) in prostate cancer leads to low genitourinary (GU) and gastrointestinal (GI) toxicity.^4^ Recently, MR‐guided radiotherapy (MRgRT) has become viable,^5^, ^6^, ^7^ resulting in even lower GU and GI toxicity.^8^ In MRgRT, a pretreatment MRI is used to delineate the clinical target volume (CTV), prior to the daily fractions of radiotherapy. At the start of each fraction, the pretreatment scan is registered to the daily fraction scan. The CTV contour is propagated by deforming it according to the registration and, if necessary, it is manually adjusted. Registration and the manual adjustment of contours are time‐consuming and hinder the effectiveness of the treatment due to potential intra‐fraction motion of the prostate.

A contour propagation method that is fast and also requires minimal manual adjustments is therefore desirable.^9^ Although the prostate is often considered to move rigidly, analyses of prostate motion have shown that a variable degree of deformation is present.^10^ For this application, deformable image registration methods are achieving very good contour propagation accuracy.^11^, ^12^, ^13^, ^14^ Conventional registration methods, however, use iterative optimization to estimate the deformation between two images, which makes these methods relatively slow, requiring several minutes.

An alternative to contour propagation is automatic segmentation of the prostate in the fraction images. In the past years, many algorithms for automated segmentation of the prostate in MR images based on deep learning have been proposed.^15^, ^16^, ^17^, ^18^, ^19^ Overall, these methods perform well at this auto‐contouring task. However, in prostate radiotherapy, prior contours are available that can be used as a basis for new contours. In addition, the CTV is not necessarily the prostate alone, and can include a variable margin around the prostate (in our case a 4‐mm margin around the GTV was used) or additional tissue (e.g., the seminal vesicles). In a propagation method, this variability in the delineations can be taken into account using a previous delineation, whereas a segmentation method would only be able to delineate the prostate, which allows for little flexibility.

Contour propagation can be interpreted as a combination of registering a moving image $I_{M}$ to a fixed image $I_{F}$ and subsequently applying the obtained transformation to the moving image’s contour $C_{M}$, which results in an estimate of the fixed image’s contour $C_{F}$. In the case of MR‐guided radiotherapy, the pretreatment scan will be the moving image, as it needs to be transformed to align with the image recorded during therapy. When applying the obtained transformation *T* to the pretreatment contour $C_{M}$, it should be similar to the contour of the fraction image $C_{F}$, that is, $C_{M}\left(T\right)$ should match $C_{F}$. Conventional image registration algorithms approach this problem as an optimization problem in which the transformation is optimized iteratively by maximizing the similarity of the images $I_{M}\left(T\right)$ and $I_{F}$.^20^ Recent studies have shown that deep learning methods can significantly accelerate deformable image registration. Unsupervised, weakly supervised, and strongly supervised neural networks have been used to estimate deformation vector fields directly from two images. *Unsupervised* methods learn the deformation directly from pairs of images without a ground truth deformation vector field by maximizing a similarity metric.^21^, ^22^, ^23^, ^24^, ^25^, ^26^, ^27^ *Strongly supervised* methods use a ground truth deformation vector field, usually by applying known transformations to a set of images during training.^28^, ^29^, ^30^, ^31^, ^32^, ^33^, ^34^ *Weakly supervised* methods are a variant of unsupervised methods, in which the similarity metric is replaced by learning an auxiliary task, such as maximizing the overlap of known segmentations.^35^ Weakly supervised registration algorithms are particularly well‐suited for contour propagation, as they implicitly can use contour overlap to guide the registration. In earlier work, we have shown that it is possible to use synthetic transformations to train a neural network for image registration in a supervised fashion in case of limited training data.^36^ In this paper, we therefore explore strongly and weakly supervised learning for contour propagation in MR‐guided prostate radiotherapy. We train a CNN to estimate deformation vector fields from two MR images and directly apply the deformation to the associated CTV segmentation. Because this can be done in one forward pass through the network, the proposed method is significantly faster compared to conventional iterative image registration. The network can be trained to optimize the deformation field, the overlap of the transformed segmentation with the true segmentation, or a combination of the two. We test three variants of the network to assess the effect of the objectives on the accuracy of the propagated contours. The network architecture that we propose is based on previous work on registration of pulmonary computed tomography (CT) inhale‐to‐exhale registration,^36^, ^37^ which showed that complex deformable registration can be accomplished end‐to‐end with supervised convolutional neural networks (CNNs). The performance of this network for pulmonary CT registration was close to that of existing conventional methods, but with substantially faster, sub‐second registration times. In this paper, we adapt this architecture to also include the transformation of the contour. We test the proposed method on the contour propagation from pretreatment to daily fraction scans. In addition, we compare to an open‐source registration method.

## 2 Materials and Methods

### 2.1 Patient data collection and preparation

**Figure 1:**
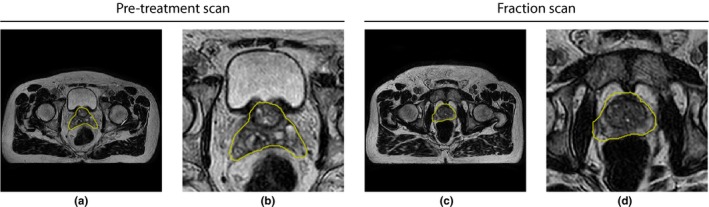
Examples of pretreatment (a) and daily fraction scans (c) with ground truth prostate contours from the same patient in yellow. Both images show the center slice of the volume. (b) and (d) show zoomed‐in versions of (a) and (c), respectively. The images are from T2‐weighted three‐dimensional (3D) turbo spin‐echo scans. [Color figure can be viewed at http://wileyonlinelibrary.com]

Data from five patients treated for prostate carcinoma on a Unity 1.5 T MR‐Linac system (Elekta AB, Stockholm, Sweden) at the UMC Utrecht hospital in the Netherlands was collected. The patients were treated between February and July of 2019, and provided informed consent for use of their data as part of the ethics review board approved MOMENTUM (Multiple Outcome Evaluation of Radiotherapy Using the MR‐Linac) study. The prescribed dose was 62 Gy, delivered in 20 daily fractions of 3.1 Gy. The images used in this study include a T2‐weighted three‐dimensional (3D) Cartesian turbo spin‐echo sequence acquired on the Elekta Unity 1.5 T system with acquisition parameters as specified in Table 1. An MRI was acquired in a pretreatment session in advance of the daily fractions to perform structure delineation and enable treatment planning. Similar MRIs were made in advance of each daily treatment session. On this so‐called pre‐beam MRI, the pretreatment contours need to be propagated such that a new plan can be generated that fits optimally to the patient’s anatomy of that given fraction. In total, 21 scans per patient were collected: 1 pretreatment scan and 20 daily fraction scans. Examples of a pretreatment scan and a daily fraction scan are shown in Fig. 1.

**Table 1:** Imaging parameters used for the acquisition of the T2‐weighted magnetic resonance images on the Elekta Unity 1.5 T system.

Parameter	Value
Sequence	3D cartesian turbo spin‐echo
Relaxation time	1535 ms
Echo time	277.8 ms
Flip angle	$90^{\circ }$
Bandwidth	740 Hz/px
Acquisition matrix^a^	268 × 268 × 44
Field of view^a^	400 × 400 × 300 mm${}^{3}$
Reconstructed voxel spacing^a^	0.83 × 0.83 × 1.0 mm${}^{3}$
Reconstructed image size^a^	480 × 480 × 300
Acquisition duration	116.7 s

a Expressed in left‐to‐right, posterior‐to‐anterior, superior‐to‐inferior.

Patients were positioned with the aid of a laser positioning system using anatomy‐based tattooed skin markers. These markers are meant to be aligned with the prostate’s axial position to enable scans with the prostate consistently centered in the field‐of‐view (FOV). At the time of the pretreatment scan, no CTV delineation has been performed, and typically an offset can occur between the marker position and the center of the prostate. After CTV delineation on the pretreatment scans, the offset is known and is accounted for in the patient setup in the daily fraction, centering the CTV in the FOV of the fraction scans. The pretreatment CTV delineation is performed shortly after acquisition. This delineation is not time‐critical given the fact that the first fraction is at least one day later than the pretreatment image acquisition.

In contrast to the delineation on the pretreatment scan, the adaptive workflow to propagate the contours to the fraction scans is time‐critical. As part of the treatment workflow, during the daily fraction, the FOV of the images was reduced in the axial direction to speed up the workflow. Specifically, the prostate contour was propagated from the pretreatment scan to the daily fraction using rigid registration, after which the image was cropped with a margin of 30 mm superior and inferior to the prostate. The rigid registration was only used to estimate the amount of required cropping, which results in a translation of the prostate in the axial direction. To obtain a similar FOV in the pretreatment images, we cropped the pretreatment images around the prostate CTV with a 30 mm margin.

An expert radiation oncologist drew gross tumor volume (GTV) contours on the pretreatment scans. The CTV was taken as a 4‐mm margin around the GTV (excluding the bladder and rectum), and further extended to include the prostate, and the seminal vesicles if they touched or had overlap with the GTV. On the daily fraction scans, CTV contours were constructed by propagation of the CTV contours from the pretreatment to the fraction scans using the ADMIRE deformable registration algorithm (Elekta AB). When necessary, the radiation oncologist manually adjusted or redrew the contours using VolumeTool, an in‐house software package.^38^ The planning target volume (PTV) was defined as the CTV with an isotropic 5‐mm margin in all directions. The manually delineated CTV contours of the pretreatment and daily fraction scans are used and considered the ground truth in this study. Each contour was converted to a binary segmentation, by assigning voxels of which the centers lie inside the contour to the segmented area.

### 2.2 Network architecture

**Figure 2:**
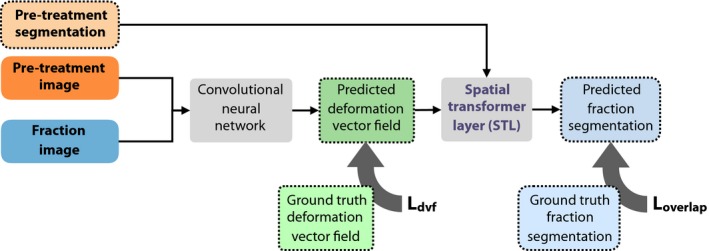
General overview of the method. The method consists of two parts. In the first part, a convolutional neural network predicts a deformation field from a pretreatment image and fraction image. In the second part, the predicted deformation field is used by a spatial transformer layer to deform the segmentation as delineated on the pretreatment image. The loss functions $L_{\mathrm{dvf}}$ and $L_{\mathrm{overlap}}$ are only computed during training. [Color figure can be viewed at http://wileyonlinelibrary.com]

A 3D CNN was designed to propagate the CTVs from the pretreatment images to the fraction images. This network consists of two parts. The first part receives the two images as two input channels and has a deformation vector field composed of three components $u_{x}$, $u_{y}$, $u_{z}$ as output. In the second part of the network, the deformation vector field is used by a spatial transformer layer (STL) to transform the prostate segmentation in the pretreatment image as delineated by the contour.^39^ The resulting transformed segmentation is the output of the second part of the network. The network can be jointly trained to optimize the transformed segmentation and the deformation vector field through a loss function that consists of two terms. The first term minimizes the $L_{2}$‐norm of the difference between the estimated and true deformation fields. The other term maximizes the Dice coefficient of the fraction segmentation and transformed pretreatment segmentation (Fig. 2).

The full loss function is defined as

(1) $$L=k_{\mathrm{overlap}}L_{\mathrm{overlap}}+k_{\mathrm{dvf}}\;L_{\mathrm{dvf}}$$

where

(2) $$L_{\mathrm{overlap}}=1-\frac{2\sum_{x\in \Omega_{F}}C_{F}\left(x\right)C_{M}\left(\hat{T}\left(x\right)\right)}{\sum_{x\in \Omega_{F}}C_{F}\left(x\right)+C_{M}\left(\hat{T}\left(x\right)\right)},$$

optimizes the overlap of the segmented volumes, and

(3) $$L_{\mathrm{dvf}}=\frac{1}{\left|\Omega_{F}\right|}\sum_{x\in \Omega_{F}}{\left\|T\left(x\right)-\hat{T}\left(x\right)\right\|}_{2}^{2}$$

optimizes the deformation vector field (DVF). $\hat{T}$ is the network’s estimate of the actual transformation **T**, and $k_{\mathrm{overlap}},k_{\mathrm{dvf}}\in \left[0,1\right]$ are weighting parameters. The focus of optimization will change based on these parameters. When $k_{\mathrm{dvf}}$ is set to zero, the network will be trained to maximize the Dice coefficient between the segmentations. No explicit deformation field is required in this case, and as a result, the deformation fields estimated by the network cannot be guaranteed to have a real physical interpretation. When we set $k_{\mathrm{overlap}}$ to zero, the network is trained to predict deformation fields similar to those in the training set. In this case, the segmentation overlap is completely ignored. The hybrid case ($k_{\mathrm{overlap}}\;=\;k_{\mathrm{dvf}}\;=\;1$) combines both training objectives.

**Figure 3:**
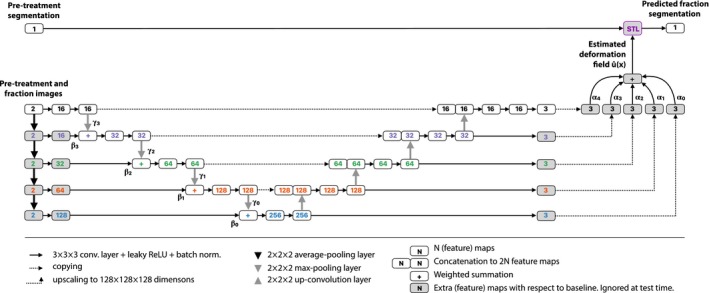
Architecture of the progressive network. The gray blocks indicate feature maps that are learned during training but ignored at test time. Compared to a typical U‐net architecture, we add input layers on the left side for every resolution level, each followed by an extra convolutional layer that matches the number of feature maps in that level. A summation node sums the output of these convolutional layers and the output of the pooling layer in the level above it. Output maps at every level are summed up weighted by ***α*** to obtain the final deformation field. This field is used to deform the input segmentation using the spatial transformer layer. [Color figure can be viewed at http://wileyonlinelibrary.com]

For the estimation of the deformation field, we use a standard three‐dimensional U‐net architecture^40^ and adapt it to accept two‐channel inputs and three‐channel outputs. The U‐net is composed of five *resolution levels* in which the convolutional layers have outputs of specific dimensions, ranging from 128 × 128 × 128 at the top of the architecture, to 8 × 8 × 8 at the bottom. Each level has a distinct color in Fig. 3. In previous work we have shown that it is beneficial to train the U‐net for image registration progressively.^34^, ^37^ To this end, we add input and output layers to each of the resolution levels. Outputs are scaled to 128 × 128 × 128 dimensions and a weighted sum is taken as the final output of the network. The weights of this sum are not learned, but set during the training process, which allows us to control which level contributes to the result. Initially, we only let the lowest dimension (8 × 8 × 8) contribute to the result. After a fixed number of iterations *N*, we linearly decrease the weight of this level to zero and linearly increase the weight of the level above it to one (16 × 16 × 16) over *M* iterations. Then we let the two‐level U‐net train for another 2*N* iterations followed by another *M* iterations of transition to a three‐level U‐net, and so on until we obtain a typical five‐level U‐net. The five‐level U‐net is then trained further in the normal way. At test time, the architecture does not differ from the typical U‐net architecture. The chosen schedule for this study used *N* = 1000 and *M* = 2000.

The output of the U‐net is a set of three maps for the $u_{x}$, $u_{y}$, and $u_{z}$ components of the deformation field. We use these maps as input to a STL that transforms the pretreatment segmentation.^39^ The STL uses nearest neighbor interpolation to sample the pretreatment segmentation based on the deformation vector field by the U‐net. The STL is differentiable, allowing the gradients of the overlap loss in Eq. (2) to backpropagate through the STL. The U‐net can map the input images to a deformation field that leads to a high overlap of the ground truth fraction segmentation and the transformed pretreatment segmentation.

### 2.3 Training set

The training set consists of pairs of synthetically deformed images and delineated prostate segmentations, for which the ground truth deformation field is generated. During each iteration of training, a unique combination of input images, segmentations, and deformation fields is constructed by applying random deformations. This allows for supervised training with a known ground truth. We hypothesize that the network should be able to generalize from these synthetic examples to real registration problems, as we have shown in previous work.^36^

**Figure 4:**
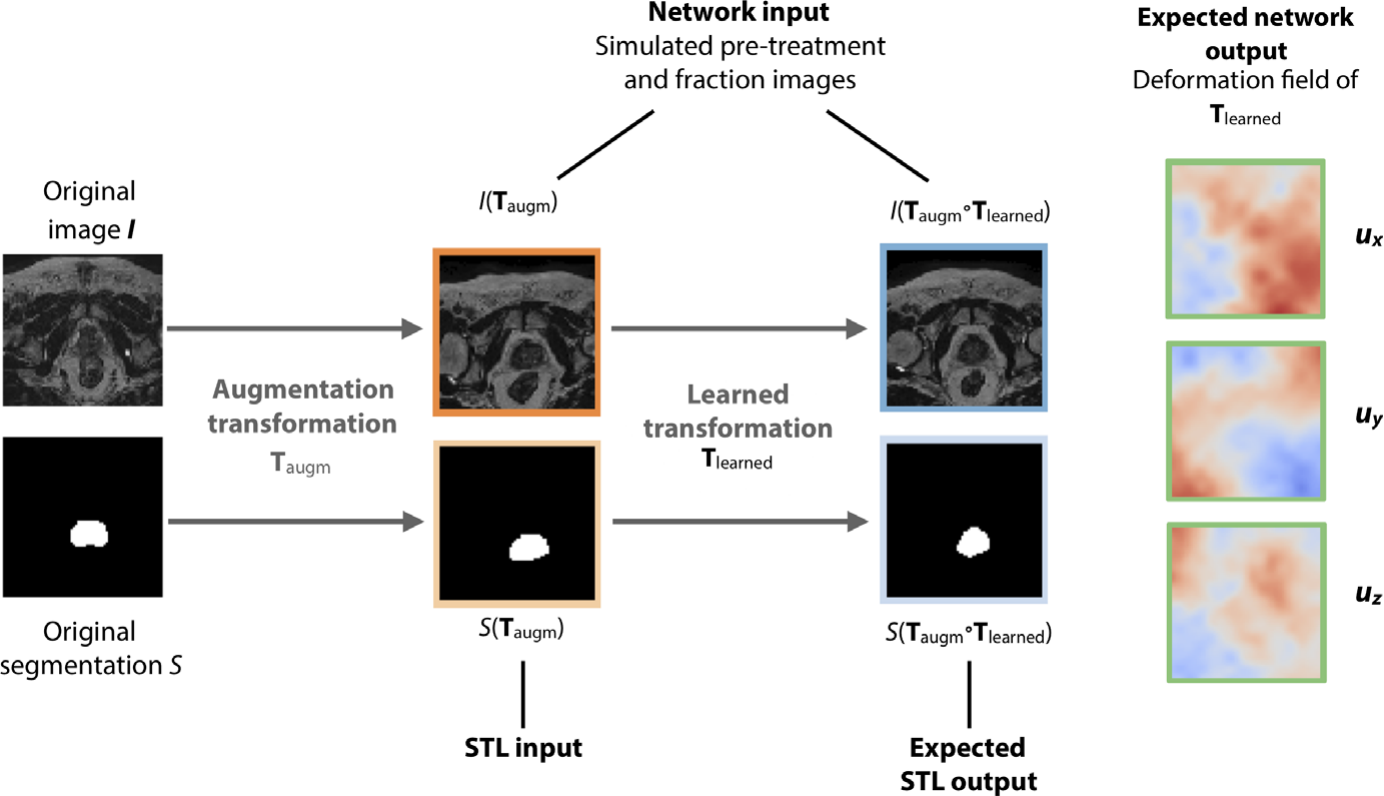
The network is trained to estimate the applied transformation $T_{\mathrm{learned}}$ from two synthetically transformed images. To increase the number of training images, an additional augmentation transformation is applied to the original data first. [Color figure can be viewed at http://wileyonlinelibrary.com]

The process of creating training examples is outlined in Fig. 4, and is similar to the proposed method for training data generation in Ref. [36, 37]. Every iteration of training, an image *I* and associated CTV segmentation *S* from the training set are selected. A random deformable transformation **T** is sampled and applied to both, resulting in *I*(**T**) and *S*(**T**). From the images *I* and *I*(**T**), the network has to predict the transformation **T** that can be applied to *S* to obtain an estimate for the ground truth segmentation *S*(**T**). To increase the amount of data, we also apply free‐form deformations for data augmentation using an additional random transformation to the images and segmentations. To limit interpolation artifacts, the augmentation transformation and the learned transformations are concatenated before they are applied to the images, and only one interpolation is required. In Fig. 4, the pair of images is therefore shown as $I(T_{\mathrm{augm}})$ and $I(T_{\mathrm{augm}}\circ T_{\mathrm{learned}})$, where $T_{\mathrm{learned}}$ is the transformation for which the network should find the deformation field, and $T_{\mathrm{augm}}$ is used for augmentation. In total, we generate 15 000 unique pairs of images and segmentations during training, for which the network is trained to estimate the deformation field.

The learned transformations consist of a sequence of random B‐spline transformations, sampled on equidistant grids of points from a uniform distribution within a specific range. The ranges and grid sizes for the concatenated transformations are shown in Table 2. The augmentation transformations consist of a random rigid transformation with rotation angles sampled from a uniform distribution between −0.1 and 0.1 radians, and translation vectors sampled from a uniform distribution between −12.8 and 12.8 voxels, followed by a random deformable B‐spline transformation similar to $t_{1}$ in Table 2. Both transformations are concatenated with a scaling transformation that effectively crops the images around the center. We scaled the images by a factor of two in‐plane, and a factor of 1.33 in the axial direction. For the images used in this study this means that the effective FOV of the network is 200 × 200 mm${}^{2}$ in the transversal directions and between 100 and 130 mm in the axial direction. Because the prostate appears larger in the images, the network can use a larger part of the estimated deformation vector field for the prostate itself. A requirement is that the prostate is positioned in the center of the FOV, which was the case for all images used in the study. The transformations are sampled on a 128 × 128 × 128 voxel grid using third‐order B‐spline interpolation. This results in the input images, the deformation field, and the output segmentation having 128 × 128 × 128 voxels, with each voxel having between a 1.56 × 1.56 × 0.86 and 1.56 × 1.56 × 1.01 mm${}^{3}$ voxel size. At training, validation, and test time, the pairs of images that are used as input to the network are preprocessed by linearly scaling the image intensities between 0 and 1. The deformation fields are expressed in voxel coordinates.

**Table 2:** B‐spline transformation parameters for the learned transformation.

Transformation	Grid size	Distribution
$t_{1}$	2 × 2 × 2	$U{\left(-6.4,6.4\right)}^{3}$
$t_{2}$	4 × 4 × 4	$U{\left(-3.2,3.2\right)}^{3}$
$t_{3}$	8 × 8 × 8	$U{\left(-1.6,1.6\right)}^{3}$
$t_{4}$	16 × 16 × 16	$U{\left(-0.8,0.8\right)}^{3}$

$U{\left(a,b\right)}^{n}$ is the multivariate uniform distribution that samples vectors with *n* components in the [*a*,*b*] interval in voxels. The resulting transformation is defined as $T_{\mathrm{learned}}\;=\;t_{1}\circ t_{2}\circ t_{3}\circ t_{4}$.

The network is optimized using stochastic gradient descent with momentum. The momentum parameter was set to 0.5, and the learning rate was set to 0.01. A batch size of one was used throughout training. All convolutional layers were trained with batch normalization, using the exponential moving average of the batch normalization parameters, as proposed by Ioffe et al.^41^

### 2.4 Experiments

To maximize the number of patients in the training set, we perform a variation of leave‐one‐out cross validation. We used the fraction scans of three patients for training, the scans of the fourth patient for validation, and the fifth patient’s scans as a test set. We run five permutations, where each patient is used once in the test set, and once in the validation set. The validation set was used to monitor the model’s performance on independent data during training. For each of the five folds, hyperparameters like the learning rate were kept the same. Each of the three networks was trained for 15 000 iterations for each of the fivefolds.

For each patient, we train three variants of the network: one trained on segmentation overlap ($k_{\mathrm{overlap}}\;=\;1$, $k_{\mathrm{dvf}}\;=\;0$), one trained on deformation field estimation ($k_{\mathrm{overlap}}\;=\;0$, $k_{\mathrm{dvf}}\;=\;1$), and a hybrid of these two ($k_{\mathrm{overlap}}\;=\;1$, $k_{\mathrm{dvf}}\;=\;1$). We do this for all instances in the cross validation. To distinguish the variants of the network, we will call them “overlap loss network,” “deformation loss network,” and “hybrid loss network” for the remainder of this paper. We compare the presented method to a more conventional deformable image registration method in Elastix, an open‐source image registration software package.^42^ We use a deformable image registration algorithm implemented in Elastix published by Klein et al.^12^ The original purpose of this algorithm was to perform automatic atlas‐based segmentation of the prostate in 3D MR images. The algorithm by Klein et al. performs a rigid registration first, followed by a deformable registration based on B‐spline transformations, by optimizing the localized mutual information.^43^, ^44^

For all network variants and Elastix, we measure the Dice coefficient of the fraction segmentation and propagated pretreatment segmentation. As a measure of registration error for the prostate, we calculate the difference between the centroid of the prostate in the propagated pretreatment segmentation and the fraction segmentation. As a metric for contour distance, we calculate the 95th percentile of the Hausdorff distance between the segmentations. We check for folding by inspecting the determinant of the Jacobian of the deformation field. As a measure of folding, we count the percentage of voxels with a negative Jacobian determinant, both for the full image and for the voxels inside the prostate. To assess the robustness of the methods, we assess how they handle additional shifts of the prostate that may occur during a fraction, by testing the performance on a range of superior–inferior shifts. These shifts are likely to occur in clinical practice due to the filling of the bladder, which is right above the prostate. We apply shifts along the axial direction between −5 and + 5 mm with 1 mm steps to each of the pretreatment images and test the propagation to all fractions for each patient. The shifts are applied by changing the cropping window that is described in Section 1.

To show the difference in speed of each of the algorithms, we record the time necessary to complete each registration problem including the transformation of the segmentation for the Elastix method and the proposed networks.

## 3 Results

**Figure 5:**
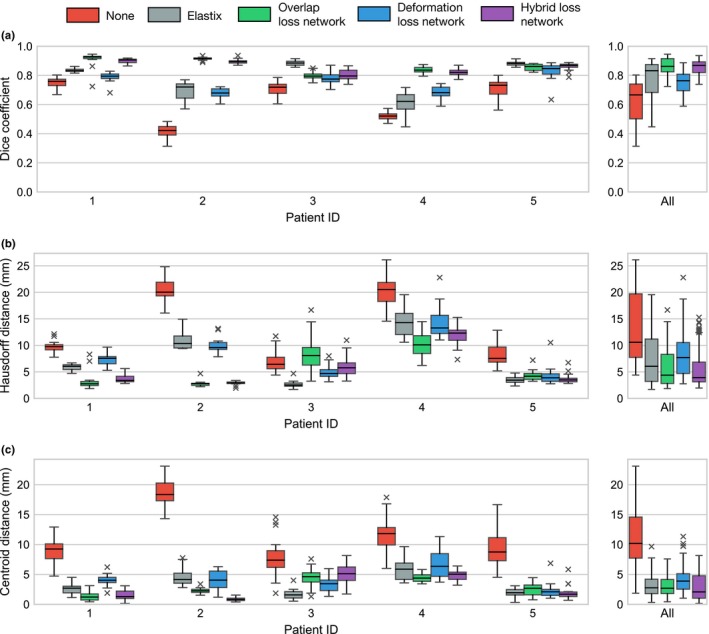
Box plots of the (a) Dice coefficients, (b) Hausdorff distances, and (c) prostate centroid distances for no registration, Elastix, and the three convolutional neural network variants per patient. Each box displays the distribution of the 20 registration problems for a single fold of the network in which that patient was in the test set. [Color figure can be viewed at http://wileyonlinelibrary.com]

In Fig. 5, we show box plots for the Dice coefficient, 95th percentile of the Hausdorff distance, and the registration error for the prostate’s centroid for the propagation from the pretreatment scan to each of the 20 fraction scans. For each patient, we show five boxes: red boxes represent the metrics without propagation, gray boxes represent Elastix’ propagation results, and green, blue, and purple boxes represent the results of the overlap, deformation and hybrid networks, respectively. On the x‐axis, boxes are grouped by patient.

**Figure 6:**
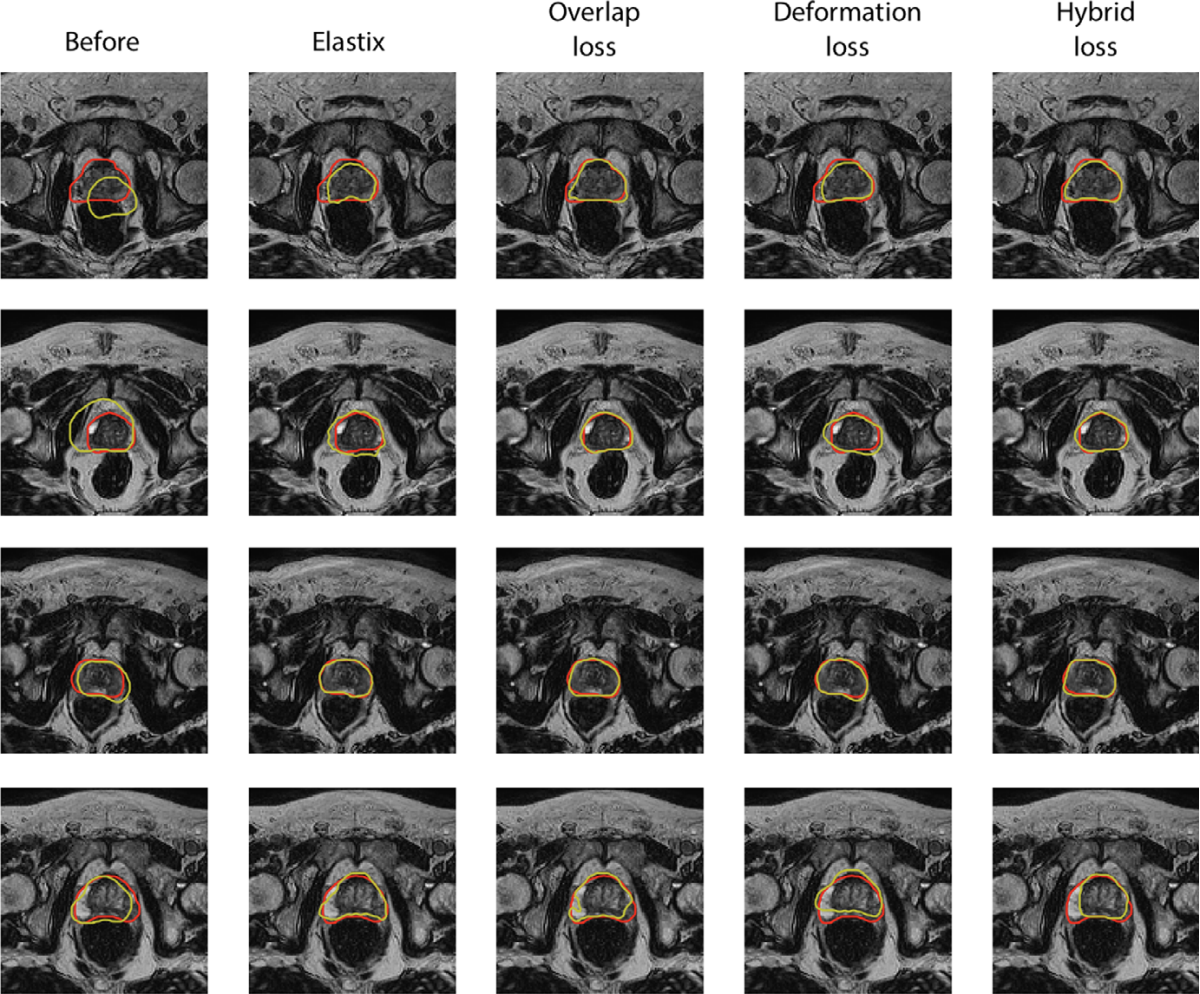
Examples of propagated contours (yellow) and ground truth contours (red). From left to right contours are shown for no propagation, the propagation by Elastix, and the propagation by the three network variants for four patients. The bottom row shows a case for which the networks fail to correctly propagate the contour. [Color figure can be viewed at http://wileyonlinelibrary.com]

In general, the results obtained by the overlap loss network and the hybrid loss network are superior to the results obtained with Elastix, with the deformation loss network having a somewhat lower Dice coefficient, and larger Hausdorff registration and centroid distances compared to the other methods (Table IV). The Wilcoxon signed‐rank test was used to analyze whether the Dice score, Hausdorff distances, and prostate centroid distances obtained by the CNNs were significantly different from those obtained by Elastix. Full results are shown in Table 3. At a significance level *α* = 0.01, we can conclude significant improvement of the Hausdorff distances and Dice coefficients over Elastix for the overlap loss and hybrid loss networks, whereas Elastix is significantly better than the deformation loss network. In Fig. 6, examples of propagated contours are shown for each of the methods, showing that generally the contours have better overlap for the overlap loss and hybrid loss networks than for Elastix and the deformation loss network.

**Table 3:** *P*‐values for the Wilcoxon signed‐rank test between Elastix and each of the three network variants.

Metric	Overlap loss	Deformation loss	Hybrid loss
Dice coefficient	$P\;<\;10^{-5}⋆$	*P* > 0.99†	$P\;<\;10^{-5}⋆$
95th perc. Hausdorff	$P\;<\;10^{-3}⋆$	*P* > 0.99†	$P\;<\;10^{-3}⋆$
Prostate centroid	*P* > 0.11∘	*P* > 0.99†	*P* < 0.05∘

Significant improvements (*P* < 0.01) over Elastix are indicated with ⋆, no significant differences with ^∘^, and cases where Elastix is superior with †.

**Figure 7:**
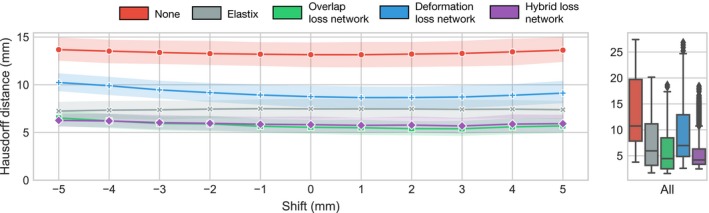
Hausdorff distance as a function of additional prostate shift, showing that the overlap loss and hybrid loss networks introduce smaller registration errors compared to Elastix. Each point is the average of the hundred registration problems (20 for each of the 5 patients). The shaded areas show the 95% confidence interval. [Color figure can be viewed at http://wileyonlinelibrary.com]

In Table 4, we show the percentage of voxels with negative Jacobian. Elastix and the deformation loss network suffer from little to no folding, whereas the networks that were also trained using the Dice coefficient show substantially more folding. Fig. 7, shows the results of the axial shifting experiment, in which the prostate is axially shifted between −5 and +5 mm. For each of the shifts, we show the distribution of the 95th percentile of the Hausdorff distance for all possible pretreatment‐to‐fraction registration problems across the five patients. The shifts induce little variation in the Hausdorff distance for any of the methods except for the deformation loss network. This indicates that the overlap loss and hybrid loss networks and Elastix are insensitive to the shifts. However, the overlap loss and hybrid loss network outperform Elastix over the whole range of applied shifts.

**Table 4:** Results of the evaluated methods in terms of Dice coefficient, 95th percentile of the Hausdorff distance, prostate centroid distance, amount of folding (Jacobian determinant < 0), and duration of each algorithm.

Metric	None	Elastix	Overlap loss CNN	Deformation loss CNN	Hybrid loss CNN
Dice coefficient	0.62 ± 0.14	0.78 ± 0.12	0.86 ± 0.05	0.75 ± 0.07	0.86 ± 0.05
95th perc. Hausdorff (mm)	13.15 ± 6.37	7.47 ± 4.72	5.82 ± 3.63	8.16 ± 4.11	5.66 ± 3.56
Prostate centroid (mm)	11.38 ± 4.78	3.29 ± 1.95	2.99 ± 1.57	4.10 ± 2.14	2.85 ± 2.04
Folding in FOV (%)	–	0.00 ± 0.00	33.97 ± 4.20	1.78 ± 0.79	7.32 ± 2.19
Duration (s)	–	43.2 ± 0.29	0.49 ± 0.10	0.49 ± 0.10	0.49 ± 0.10

Each figure is the mean ± standard deviation over the 20 pretreatment‐to‐fraction propagations averaged over all patients.

For each of the methods, we recorded the time required to complete the propagation of the segmentation on a system with an Intel Xeon E5‐2640 v4 CPU, 512 GB of memory and an Nvidia Titan XP graphics card with 12 GB of GPU memory. For each of the network variants, the timing is the same, amounting to 0.49 ± 0.10 s (*μ* ± *σ*) when running on the GPU. For Elastix, executing the propagation problem required on average 43.2 ± 0.29 s, using both the CPU and GPU for the registration.

## 4 Discussion

In this paper, we have proposed a deep learning‐based method for fast deformable propagation of clinical target volume contours from pretreatment to fraction scans. Three variants of a CNN trained with different loss functions have been tested, based on contour overlap, prediction of the deformation field, and a hybrid of the two.

The results show that the propagation accuracy of the networks trained with the overlap loss and hybrid loss perform significantly better (*P* < 0.001) at Dice coefficients and Hausdorff distances compared to the open source registration package Elastix. Hausdorff distances measured between the predicted contours and the ground truth indicate that the registration error at the contours is on average 5.7 mm for the hybrid loss network compared to 7.5 mm for Elastix. The deformation loss network on average performs worse than the other two network variants and Elastix. A crucial aspect for the application is timing: the networks can propagate the prostate segmentations within 0.5 s, much faster than the 43 s it takes to perform the propagation in Elastix.

Inspection of folding in the predicted deformation fields shows that the deformation loss network and Elastix perform much better in this respect, with less than two percent of the FOV showing any folding. The deformation loss network will cause the network to mimic the transformations in the training set, which do not fold by construction. Because the overlap loss and hybrid loss networks maximize prostate overlap, the learned deformation fields can contain unrealistic deformations. It should be noted that for the current application the true deformation field can show folding as well, as sliding motion between the prostate and surrounding organs is possible.

A shift experiment shows that the overlap and hybrid loss networks are not very sensitive to larger displacements of up to an additional 5 mm between the prostate in the pretreatment and fraction scan. The fraction scans used in this study show that it is possible to position the patient accurately based on the tattooed markers, and that expected displacements between fraction scans falls within this range. The network trained on deformation loss was shown to be more sensitive to additional shifts, which can be attributed to limitations in the distribution of deformations in the training set.

Our results show that it is possible for the networks to generalize from a set of four patients (three in the training set, one in the validation set) to a fifth patient. This also suggests that with additional data becoming available, it will be possible to train a single neural network that may generalize to new patients. To cope with the currently limited number of patients, we have adopted a leave‐on‐out strategy. With more data becoming available, it will ultimately be possible to train a single model that can be used for multiple new patients.

The proposed method introduces two hyperparameters that need to be optimized, $k_{\mathrm{overlap}}$ and $k_{\mathrm{dvf}}$, that weight high prostate overlap and correct estimation of the deformation vector field respectively. In the experiments we have tried binary settings of these parameters, and there is potentially a better, non‐binary setting for the current application. With the limited data available, we have not been able to test this, but future work could focus on the optimization of the hyperparameters.

Delineation methods using deep learning based prostate segmentation can also be employed to delineate CTVs on fraction scans, provided the CTV always covers the same type of tissue, as discussed in the introduction section. Segmentation methods perform well when the CTV covers only the whole prostate, but fail when other surrounding tissue should be included in the CTV. A CNN trained to propagate contours can account for these variations in the pretreatment delineations, by using information from previous contours. Although a quantitative comparison to prostate segmentation is difficult because of these differences, the Dice coefficients and Hausdorff distances for the two best performing networks are in the same range as deep learning‐based prostate segmentation methods in literature.^15^, ^16^, ^17^, ^18^, ^19^ However, it should again be noted that prostate segmentation is not the same as CTV segmentation in this case.

Further applications of the proposed method include the propagation of multiple contours at once. The proposed networks can be adapted to transform multiple segmentations using the STL. In that case, the setup stays the same, except for the fact that multiple masks are fed to the STL, and that the overlap loss will need to be replaced with the multi‐class generalized overlap loss.^45^ The transformation of multiple segmentations can happen in parallel on the GPU, which means that there will be no additional computation time for multiple contours. The proposed method may also be valuable in *intra*‐fraction propagation of contours, by registering the pretreatment or a previous fraction scan to cine‐MR images that are continuously acquired during MR‐guided treatment.^46^ This is particularly of interest for hypofractionated schemes, in which the individual fractions may take more time, which can potentially increase the impact of registration errors during the treatment. To measure the efficacy of the propagated contours, future work will include a more extensive validation investigating the time required for a specialist to adapt the contours.

It is important to note that the adaptive MRgRT workflow is performed under continuous human supervision and that this would also be the setting in which the current method would potentially be used. The radiation oncologist will always check the contours and improve them manually where necessary. A further speed improvement can be obtained by reducing the manual corrections required from the radiation oncologist.

## 5 Conclusions

We have developed a fast contour propagation method for prostate radiotherapy, that can propagate contours from pretreatment to fraction scans in under half a second. The method achieves superior propagation accuracy to an existing and more time consuming deformable registration method. The proposed method can aid in shortening treatment time by reducing time spent on propagation and manual contour adaptation.

## Conflict of Interest

The authors have no conflict to disclose.
